# Increased Neutralizing Antibody Production and Interferon-γ Secretion in Response to Porcine Reproductive and Respiratory Syndrome Virus Immunization in Genetically Modified Pigs

**DOI:** 10.3389/fimmu.2017.01110

**Published:** 2017-09-12

**Authors:** Guangping Huang, Xianyong Liu, Xiaoli Tang, Li Du, Wenhai Feng, Xiaoxiang Hu, Liangquan Zhu, Qiuyan Li, Xun Suo

**Affiliations:** ^1^State Key Laboratory of Agrobiotechnology, College of Biological Sciences, China Agricultural University, Beijing, China; ^2^College of Veterinary Medicine, China Agricultural University, Beijing, China; ^3^Key Laboratory of Animal Epidemiology of the Ministry of Agriculture, Beijing, China; ^4^Department of Microbiology and Immunology, College of Biological Sciences, China Agricultural University, Beijing, China; ^5^China Institute of Veterinary Drug Control, Beijing, China

**Keywords:** neutralizing antibody, interferon-γ, antiviral immune responses, CD28, transgenic pig

## Abstract

T cell-mediated immunity plays a prominent role in combating pathogens infection. Both the engagement of the T cell receptor with the peptide-bound major histocompatibility complex and a costimulatory signal are needed for the complete activation of the T cell. To determine whether host immune responses to vaccination could be improved by enhancing CD28-mediated costimulation and verify whether the boosted immune responses could protect the host against viral challenge, we produced a transgenic pig line expressing an extra copy of the CD28 gene controlled by its own promoter at the *Rosa26* locus. As expected, in response to porcine reproductive and respiratory syndrome virus (PRRSV) strain vaccination, CD4^+^ T cells was remarkably increased in CD28 transgenic pigs and a similar response in CD8^+^ T cells was elicited after challenge. Importantly, because of increased T cell frequencies, the virus-neutralizing antibody against JXA-1 (a highly pathogenic Chinese PRRSV strain), as well as interferon-γ secretion, were enhanced in transgenic pigs. These findings in our translational study provide a novel concept for farm animal breeding in disease resistance, in which we may use the transgenic technology to force overexpression of confirmed immunity-promoting molecules like CD28 and produce an animal with enhanced immune responses to vaccination and broad-spectrum resistance to infectious diseases.

## Introduction

There is growing evidence that T cells are central effectors for adaptive immune responses, which help protect the host against pathogens infection (1–3). In order to perform their function, T cells need to be activated, a process that requires two signals for their optimal function (4–6). One is an antigen-specific signal derived from the peptide-bound major histocompatibility complex (MHC) interaction with the T cell receptor (2, 4, 5, 7, 8). The other signal is delivered by the binding of costimulatory molecules to their ligands (2, 6, 7). It is well known that signaling solely through the interaction of TCR with MHC–peptide complex results in anergy (9). Costimulatory molecules, playing a key role in the modulation of T cell responses, are becoming promising candidates for immunotherapy (10). Among them, CD28 provides a more robust costimulatory signal, which has been well documented in numerous studies (11–13).

CD28, a homodimeric stimulatory cell membrane receptor of the immunoglobulin superfamily, is expressed on almost all CD4^+^ T cells; on nearly half of CD8^+^ human T cells and on virtually all murine T cells (14–16). When engaged with its ligand B7 molecule, which is expressed on activated B cells, dendritic cells or macrophages, CD28-mediated costimulatory signals initiate activation of AKT, which subsequently results in activation of the downstream targets nuclear factor-κB (NF-κB), nuclear factor of activated T cells, and others (17). In addition, CD28 also induces activation of RAS and the downstream phosphorylation of AKT, c-Jun N-terminal kinases, and extracellular signal-regulated kinases (ERKs), enabling high-level production of IL-2 and effective T cell proliferation and differentiation (6, 18).

Previous studies have indicated that the primary T cell response can proceed in the absence of CD28 but requires a strong or long-lasting TCR signal (11). This suggests that CD28 costimulation is necessary for the amplification of TCR signaling. However, CD28 expression is not static, as its level increases following T cell activation while the inducible expression of cytotoxic T lymphocyte-associated antigen 4 on activated T cells can downregulate CD28 levels (6, 12). Hence, there are some controversies over whether CD28 is required for maintaining T cell responses following activation.

Recent studies have verified that CD28 costimulation substantially affects immune responses. For instance, CD28^flox/flox^OX40^cre/+^ mice in which CD28 is lost after T cell priming showed fewer Th1 cells and impaired formation and maintenance of T follicular helper cells after influenza virus infection (19). More importantly, for T cell memory responses, global removal of CD28-through blockade with antimouse CD28-specific antibodies or inducible deletion by tamoxifen treatment reveals its requirement for full clonal expansion and effector functions such as degranulation or interferon-γ (IFN-γ) production during the secondary immune response to *Listeria monocytogenes* (20). These findings show that CD28-mediated costimulation plays a critical role in the induction of effective T cell immune response. However, little is known about the effects of CD28 signaling on the enhancement of immune responses in pigs.

Porcine reproductive and respiratory syndrome (PRRS) is one of the most economically devastating diseases that severely threaten swine production worldwide, leading to reproductive failures in pregnant sows and respiratory problems that persistently infect offspring piglets (21–23). The causative agent of this disease, PRRS virus (PRRSV), first identified in Europe and the United States in the early 1990s, has been spreading and threatening the global swine farming (24). To date, although commercial vaccines against PRRSV are available, they fail to provide efficient protection against the virus variable strains, in particular against highly pathogenic PRRSV (HP-PRRSV) strains (25). However, the weak T cell response to PRRSV is a particular feature of this host–virus interaction (26). Therefore, enhancement of T cell function would be an ideal measure not only for disease control but also for improving vaccine efficacy.

In the current study, to determine whether enhancing the porcine CD28 signaling could increase the host immune response to PRRSV infection, we first produced transgenic pigs expressing an extra copy of the porcine CD28 gene and then analyzed the protective effect of the immune response elicited by vaccination against the PRRSV challenge.

## Materials and Methods

### Ethics Statements

All animal experiments were performed in strict accordance with the Guide for the Care and Use of Laboratory Animals of the Ministry of Science and Technology of China and were approved by the Institutional Animal Care and Use Committee of China Agricultural University (certificate of the Beijing Laboratory Animal employee, ID: 18086). All efforts were made to minimize animal suffering.

### Animals and PRRSV Strain

F0 generation CD28 transgenic landrace pigs were produced by somatic cell nuclear transfer (SCNT). Briefly, primary porcine fetal fibroblasts isolated from a 30-day fetus were cotransfected with 4 µg of linearized pCDOCNDR and 1 µg of pCas9-sgRNA. After a 10-day selection with G418 (in concentration ranges of 500 to 1,000 µg/mL) and DTA negative selection, the cell clones were subjected to PCR analysis for the integration of CD28 at the *Rosa26* locus. Positive clones were used as donor cells and SCNT was performed as described previously (27). JXA1-R, a modified live Chinese HP-PRRSV vaccine, was a kind gift from China Animal Husbandry Industry Co. Ltd., Beijing, China. JXA-1 (GenBank ID: EF112445.1), one of the earliest Chinese HP-PRRSV strains, was the parental strain of JXA1-R, and propagated and titrated on porcine alveolar macrophages as previously described (28).

### Southern Blot

For the detection of the correct integration of CD28 into the *Rosa26* locus, 10 µg of genomic DNA were extracted from the tail samples of transgenic (Tg) and wild-type (WT) pigs, digested with *Not*I overnight and then resolved by 1% agarose gel electrophoresis. DNA samples were transferred onto a nylon membrane and then hybridized with a digoxigenin (DIG)-labeled probe amplified with the primers P1 and P2. Then they were incubated with anti-DIG AP (Cat#1093274, Roche), and the location of the probe was detected by chemiluminescent methods as described previously (29).

### Western Blot

For the detection of CD28-Flag expression, peripheral blood lymphocytes (PBLs) were lysed with RIPA buffer (10 mM Tris, pH 7.4, 150 mM NaCl, 0.2% Triton X-100, 2 mM EDTA, 1 mM PMSF, and 1× protease inhibitor mixture) and then separated on 15% polyacrylamide gels. Separated proteins were transferred to nitrocellulose membranes (Amersham Pharmacia, UK), blocked with 5% skim milk in PBST at room temperature for 1 h, and then detected with anti-Flag mouse monoclonal antibody (Sigma-Aldrich F1804). Anti-β actin antibody was used as an internal reference. Next, proteins were incubated with goat antimouse IgG-HRP (ab97023; Abcam) for 1 h at room temperature and washed three times with in PBST. The membranes were subjected to luminol-based chemiluminescence with a commercial substrate (Millipore, USA) and Kodak film.

Since ERK1/2 mitogen-activated protein kinase pathway transduces extracellular signals into intracellular responses in mammalian somatic cells (30), we evaluated the expression of phosphorylated (p)-ERK1/2 in PBLs of WT and Tg pigs. We used anti-p-ERK1/2 (9101, Cell Signaling) and anti-total-ERK1/2 (4695, Cell Signaling) antibodies at 0, 30, 60, and 90 min after stimulation with antipig CD3 mAb (5 µg/mL, clone PPT3, Abcam) and soluble antipig CD28 (2 µg/mL, prepared by National Animal Protozoa Laboratory, Beijing, China). Anti-GAPDH antibody (14C10, Cell Signaling) was used as an internal reference.

### Indirect Immunofluorescence Assay (IFA)

PBLs from Tg and WT pigs were subjected to IFA. Briefly, permeabilized and blocked cells were incubated with anti-FLAG monoclonal antibody (F1804, Sigma-Aldrich) and then with the goat antimouse IgG H&L (Cy3) secondary antibody (ab97035, Abcam, Cambridge, UK). Nuclei of cells were stained with 4′,6-diamidino-2-phenylindole (Sigma-Aldrich), and then slides were observed under a fluorescence microscope (Leica TCS SP5, Leica Microsystems, Germany).

### RNA Extraction and Quantitative Real-time PCR

For the detection of the transcription levels of CD28, total RNA from PBLs of Tg or WT pigs was extracted using TRIzol reagent^®^ according to the instructions of the manufacturer (Invitrogen, Carlsbad, CA, USA). All RNA samples were DNase-treated prior to cDNA synthesis. Total RNA was reverse-transcripted using an EasyScript First-Strand cDNA Synthesis SuperMix (Transgen, Beijing, China). Primers for real-time PCR are shown in Table 1. For PCR amplifications, 1 µL of cDNA was added to a mixture containing 10 µL of 2 × SYBR green master mix (Takara, Dalian, China), 0.4 µL of ROX reference dye, and 0.4 µL of each primer P7 and P8 (50 pmol/μL). All samples were tested in duplicate. At the end of the amplifications, cycle threshold (Ct) values were obtained. Transcripts of porcine GAPDH were used as internal reference. Relative transcript levels were quantified by the 2^−ΔΔCT^ method as described previously (31).

**Table 1:** Primer pairs used for conventional PCR and real-time PCR.

Primer	Sequences (5′–3′)	Amplicon size (bp)
P1	ATGATCCTCGGGTTACTCCTG	501
P2	GCTATAGAAAGCTACGACTCC	
P3	CGCACCCTTACCTTGTCCCA	2,500
P4	GAAGGTGGGATGGAGGGTGA	
P5	ACCGCTTCCTCGTGCTTTAC	8,000
P6	AGCTGCCTCCTGTGATTACC	
P7	AACAGTGACATTCTACCTCC	146
P8	CTGGACAATGTTTCTCTTTCAC	
P9	ACTCTCTGCACTCACGGAAGGTG	127
P10	GGCGTTTCGCATCTTAAACGGC	

For the evaluation of the viremia of PRRSV-infected WT and Tg pigs, viral RNA was extracted from serum samples collected at day 10 postchallenge with TRIzol^®^ LS reagent (Invitrogen, Carlsbad, CA, USA). The plasmid containing the non-structural protein gene of PRRSV was used to construct the standard curve. The levels of viral load in sera of Tg and WT pigs were determined with primers P9 and P10 by using absolute quantitative real-time PCR as previously described (32).

### Enzyme-Linked Immunosorbent Assay (ELISA)

Serum was separated from clotted blood and preserved at −80°C until use. PRRSV antibody levels in serum were measured using the IDEXX PRRS X3 ELISA Kit (IDEXX Laboratories HerdCheck Switzerland AG) following manufacturer’s instructions. The presence or absence of antibody to PRRSV was determined by calculating the sample to positive (S/P) ratio for each sample. An S/P ratio of 0.40 or greater was considered seropositive (26). Anti-IFN-γ antibody in serum samples was determined using Pig IFN gamma ELISA Kit (ab113353, Abcam). All ELISA procedures were performed as per the manufacturer’s instructions. Each sample was assayed in triplicate.

### Flow Cytometry Analysis

Swine peripheral blood mononuclear cells (PBMCs) were cultured in 24-well round bottom plates (1 × 10^6^ cells/well) and stimulated for 12 h with PRRSV antigen, recovered by centrifugation and resuspended in 100 µL of PBS containing 10% porcine serum. After washing twice with a cell-staining buffer (BD Biosciences), the cells were suspended in 50 µL of staining buffer and stained with PE-Cy™ 5.5 antipig CD3ε (clone BB23-8E6-8C8, BD Biosciences), APC antipig CD4 (clone 74-12-4, BD Biosciences) and FITC antipig CD8 (clone 76-2-11, BD Biosciences) at 4°C for 30 min. Following another two washes, the cells were suspended in 200 µL of sterile PBS and analyzed using a BD Accuri™ C6 flow cytometer.

### Virus Neutralization (VN) Assay

The PRRSV neutralizing antibody response in serum was measured using a modified fluorescent focus neutralization assay (33). Briefly, twofold serial dilutions (1:4 to 1:256) of heat-inactivated serum samples were prepared in 96-well plates using Minimum Essential Medium (MEM, GIBCO^®^ brand; Life Technology, Grand Island, NY, USA) supplemented with 10% FBS. An equal volume (50 µL) of PRRSV (HP-PRRSV strain) at a concentration of 2 × 10^2^ TCID_50_/mL was added to each sample, incubated for 1 h at 37°C and transferred to a 96-well plate containing confluent pulmonary alveolar macrophages (PAMs). After 24 h, the plates were washed, fixed in ice-cold 80% acetone and stained with mouse anti-PRRSV nucleocapsid monoclonal antibody diluted 1:5,000 in PBS. Cells were incubated with FITC-conjugated goat antimouse IgG-HRP (ab97023; Abcam) for 1 h at room temperature. The neutralizing activity of each serum was reported as the reciprocal of the highest dilution that caused a 90% or greater reduction in the number of fluorescent foci (34). Triple independent repeats were performed in this study.

### ELISPOT

Interferon-γ ELISPOT was performed as previously described (26). Briefly, 96 wells of ELISPOT plates were coated with antiporcine IFN-γ monoclonal antibody (5 µg/mL) (Mabtech, Mariemont, OH, USA). 5 × 10^5^ PBLs from PRRSV-vaccinated Tg and WT pigs in 100 µL RPMI 1640 complete medium were added to each well. Cells were stimulated with CD4^+^ (M6_aa33–47_: ALKVSRGRLLGLLHL) and CD8^+^ (Nsp5_aa149–167_: LHNMLVGDGSFSSAFFLRY) T cell epitopes of PRRSV (prepared by China Animal Husbandry Industry Co., Ltd., Beijing, China) (35). The wells received an equal amount of bovine serum albumin-coated beads or 2 µg/mL concanavalin A (ConA), which were used as negative and positive controls, respectively. Cells were cultured for 24 h at 37°C in 5% CO_2_. The plates were then washed five times with PBS (200 µL/well). Captured IFN-γ was detected with biotinylated antiporcine IFN-γ antibody P2C11 and streptavidin-ALP. All procedures were conducted according to the instructions of the manufacturer (Mabtech, Mariemont, OH, USA).

### Experimental Design

To test whether the upregulated CD28 costimulation could protect the piglets from the PRRSV challenge, five male Tg and five male WT pigs 4 weeks old were treated and housed in separate pens. All pigs were confirmed sera-negative for anti-PRRSV antibodies by ELISA and PRRSV-free in blood by real-time qPCR as described above. On day 0, all pigs were immunized intramuscularly (IM) with a single dose of JXA1-R vaccine. On day 28 postvaccination, all pigs in each group were challenged with 50-time doses of JXA1-R vaccine, and monitored daily for rectal temperature during the first seven days after challenge. Body weight was measured on days 0 and 28 postvaccination and day 10 postchallenge. Blood samples were collected on days 0, 14, and 28 postvaccination and day 10 postchallenge.

### Statistical Analysis

SPSS 19.0 software was used for statistical analysis. Data were expressed as mean ± SEM. Statistical significance was determined by Student’s *t*-test and *P* values <0.05 were considered significantly different.

## Results

### Generation and Identification of CD28 Transgenic Pigs

**Figure 1:**
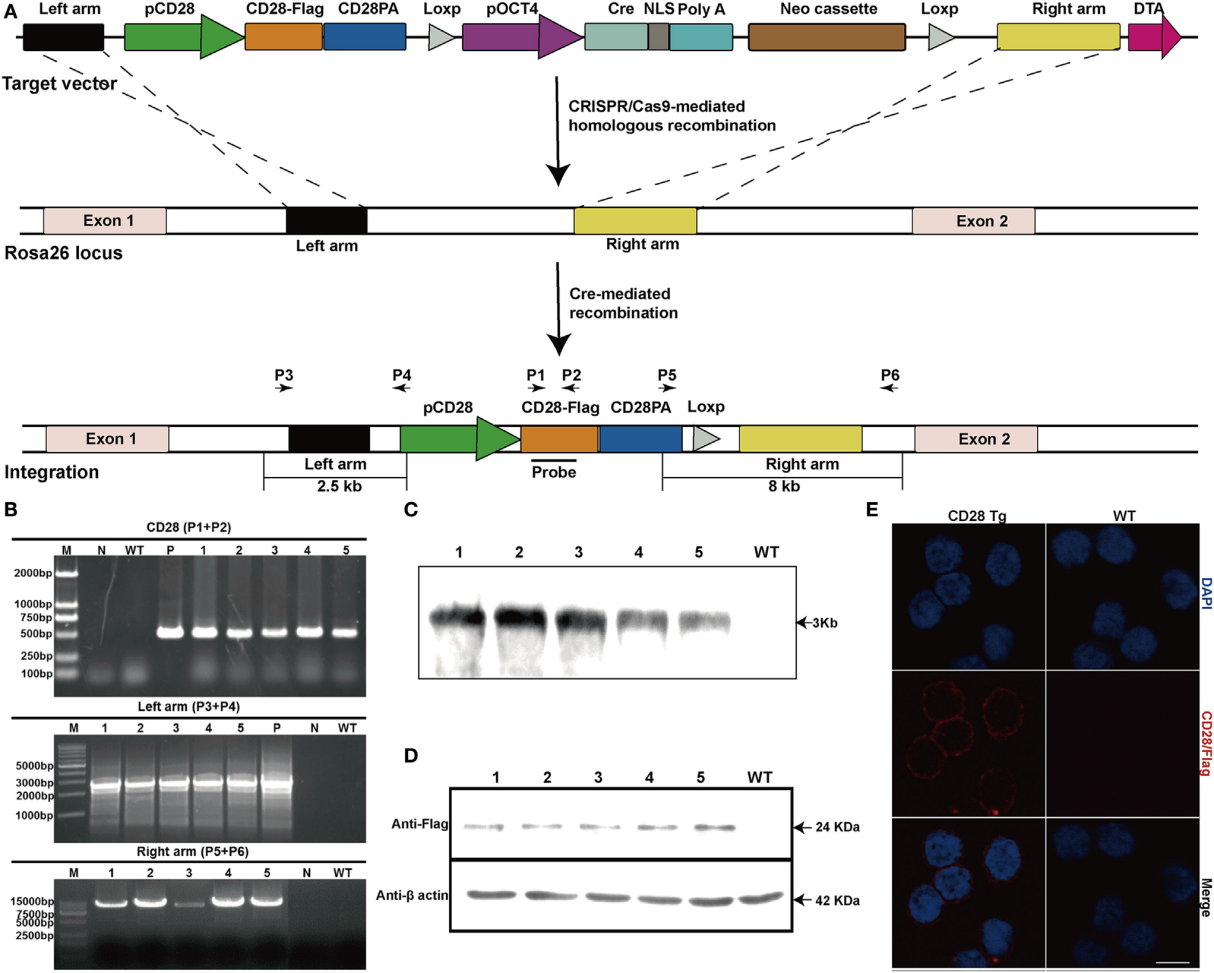
Production and identification of CD28 transgenic pigs. **(A)** Schematic diagram of genome editing strategy to target the *Rosa26* locus in *Sus scrofa* via CRISPR/Cas9-mediated homologous recombination. The black arrows indicate the primers used for PCR assay (pCD28, CD28 promoter; CD28-Flag, CD28 coding sequence fused with Flag tag; CD28PA, CD28 3′ untranslated region; pOCT4, OCT4 promoter; NLS, nuclear localization sequence). **(B)** PCR analysis of transgenic piglets using the indicated primers (1–5, genomic DNA from transgenic piglets; N, water; P in top, plasmid pCDOCNDR used as positive control; P in middle, transgenic fibroblasts used as a positive control). **(C)** Southern blot analysis of transgenic pigs (WT, genomic DNA from wild-type pigs). **(D)** CD28-Flag expression analysis by Western blotting (1–5, five transgenic pigs; WT, wild-type pig). Data are representative of three independent Western blotting experiments. **(E)** Representative immunofluorescence images showing the expression of CD28-Flag on the cell membrane of peripheral blood lymphocytes from transgenic pigs. Scale bar, 5 µm.

A correctly modified porcine fetal fibroblast cell clone obtained from the G418-resistant cell clone was used as a donor cell line for SCNT. Two out of four surrogate mothers became pregnant, and five cloned piglets were born 114 days later. PCR analysis showed that the sizes of amplicons were 0.5 kb with primers P1 and P2, 2.5 kb with primers P3 and P4 and 8 kb with primers P5 and P6 (Figures 1A,B), revealing that another copy of the CD28 gene was integrated into the *Rosa26* locus via CRISPR/Cas9-mediated site-specific homology recombination. Site-specific insertion of the CD28 gene was also confirmed by Southern blot analysis, and a single 3 kb DNA band indicated correct targeting (Figure 1C). CD28-Flag was expressed on PBLs from all the cloned piglets (Figures 1D,E). Notably, all Tg piglets developed normally and no significant differences were shown in complete blood count and blood routine parameters between Tg and WT piglets (Table 2).

**Table 2:** Serum hematological and biochemical parameters of wild-type and CD28 transgenic pigs.

Items	WT	CD28 Tg	Significant (*P* value)
RBC (10^12^/L)	7.35 ± 0.13	7.42 ± 0.09	0.70
HGB (g/L)	123.00 ± 3.06	117.30 ± 3.18	0.27
HCT (%)	39.43 ± 0.66	37.83 ± 0.78	0.19
PLT (10^9^/L)	333.00 ± 13.58	312.00 ± 16.56	0.38
WBC (10^9^/L)	19.30 ± 2.46	21.40 ± 1.42	0.50
MCH (pg)	16.70 ± 0.15	16.13 ± 0.18	0.07
MCHC (g/L)	311.70 ± 2.67	310.30 ± 3.53	0.78
GLU (mmol/L)	4.82 ± 0.08	4.65 ± 0.07	0.17
TP (g/L)	71.22 ± 2.37	73.68 ± 2.08	0.48
ALT (U/L)	56.45 ± 2.39	60.50 ± 7.32	0.63
AST (U/L)	35.57 ± 2.79	33.99 ± 1.85	0.66
UN (mmol/L)	6.13 ± 0.14	6.15 ± 0.15	0.91
Ca (mmol/L)	2.64 ± 0.03	2.63 ± 0.03	0.76
P (mmol/L)	2.65 ± 0.06	2.58 ± 0.06	0.41

*P < 0.05 was considered statistically significant*.

*RBC, red blood cell; HGB, hemoglobin; HCT, red blood cell-specific volume; PLT, blood platelet; WBC, white blood cell; MCH, mean corpuscular hemoglobin; MCHC, mean corpuscular hemoglobin concentration; GLU, glucose; TP, total protein; ALT, alanine aminotransferase; AST, aspartate transaminase; UN, urea nitrogen; Ca, calcium; P, phosphorus*.

### Increased CD28 Expression Enhances Phosphorylation of the Downstream Kinase during T Cell Activation

**Figure 2:**
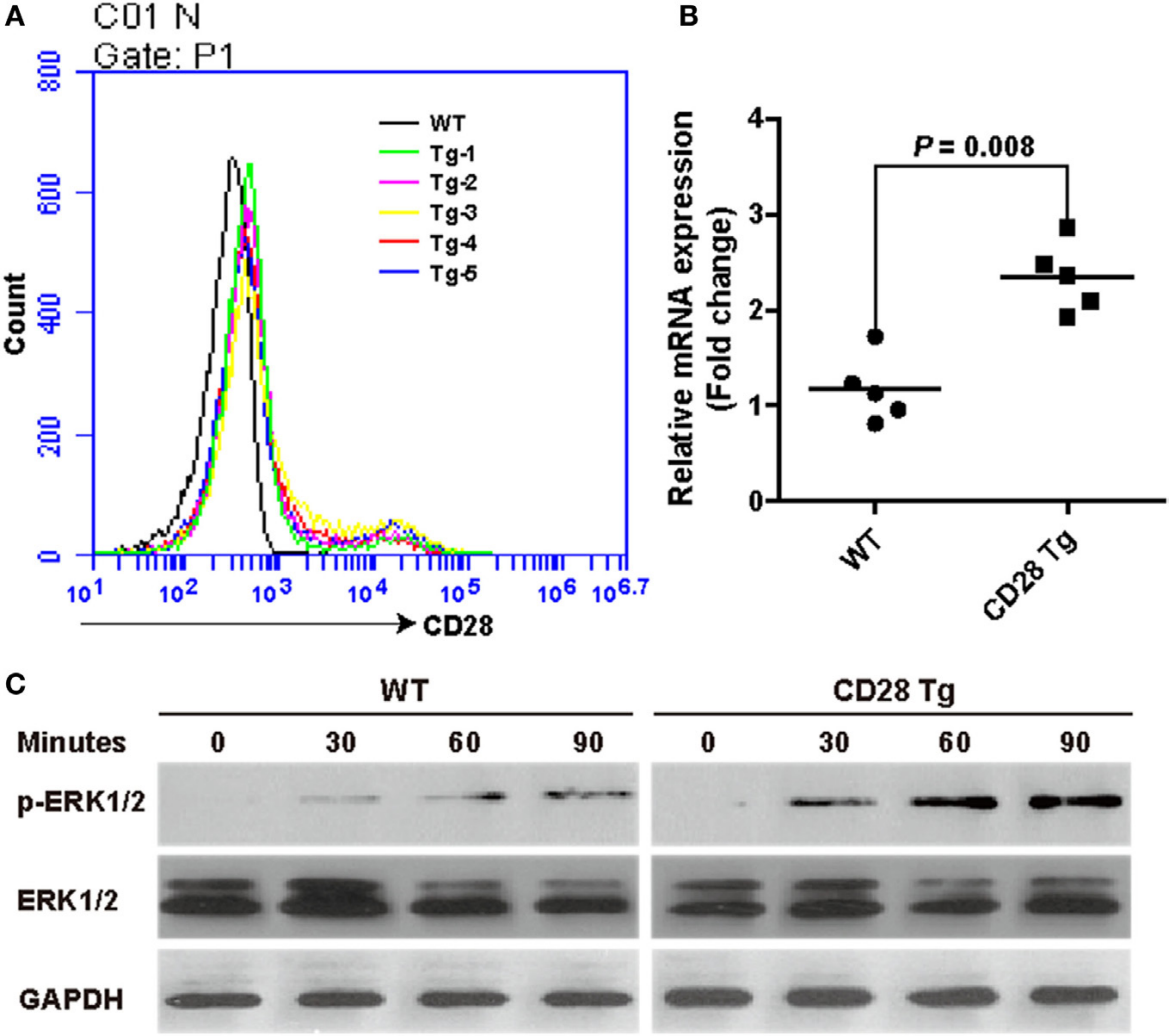
Incremental CD28 expression in transgenic pigs increases phosphorylation of the downstream kinase extracellular signal-regulated kinases (ERK) 1/2 during T cell activation. **(A)** The mean fluorescence intensity of the CD28 gene was measured by flow cytometry (WT, wild-type pigs; Tg-1–5, five transgenic pigs). **(B)** Real-time PCR analysis of the CD28 mRNA levels in transgenic (CD28 Tg) and WT pigs. **(C)** Western blot analysis of the phosphorylation of downstream kinase ERK1/2 in transgenic (CD28 Tg) and WT pigs. Data are representative of three independent Western blotting experiments with five animals per group (transgenic and wild-type groups). *P* < 0.05 was considered statistically significant.

To further verify the efficient expression of CD28 at the *Rosa26* locus, mean fluorescence intensity of CD28 was analyzed between WT and Tg pigs. Remarkably, transgenic T cells showed a nearly one-fold increase in CD28’s mean fluorescence intensity (956 ± 39 in WT vs. 1,655 ± 73 in Tg), consistent with CD28 mRNA expression levels (Figures 2A,B). Additionally, to determine the effect of CD28 overexpression on T cell activation, a downstream target kinase, ERK1/2, which is involved in TCR/CD28-mediated T cell activation, was analyzed by Western blotting. The results show that joint ligation of CD3 and CD28 with anti-CD3 plus anti-CD28 mAbs strongly synergized to phosphorylate the ERK1/2 kinase (Figure 2C), demonstrating that overexpression of CD28 contributed to the increase of phosphorylation events during T cell activation.

### CD28 Tg Pigs Show Improved Specific Antibody Response and a Reduction in Body Weight Loss, Clinical Fever, and Viral Load during PRRSV Infection

**Figure 3:**
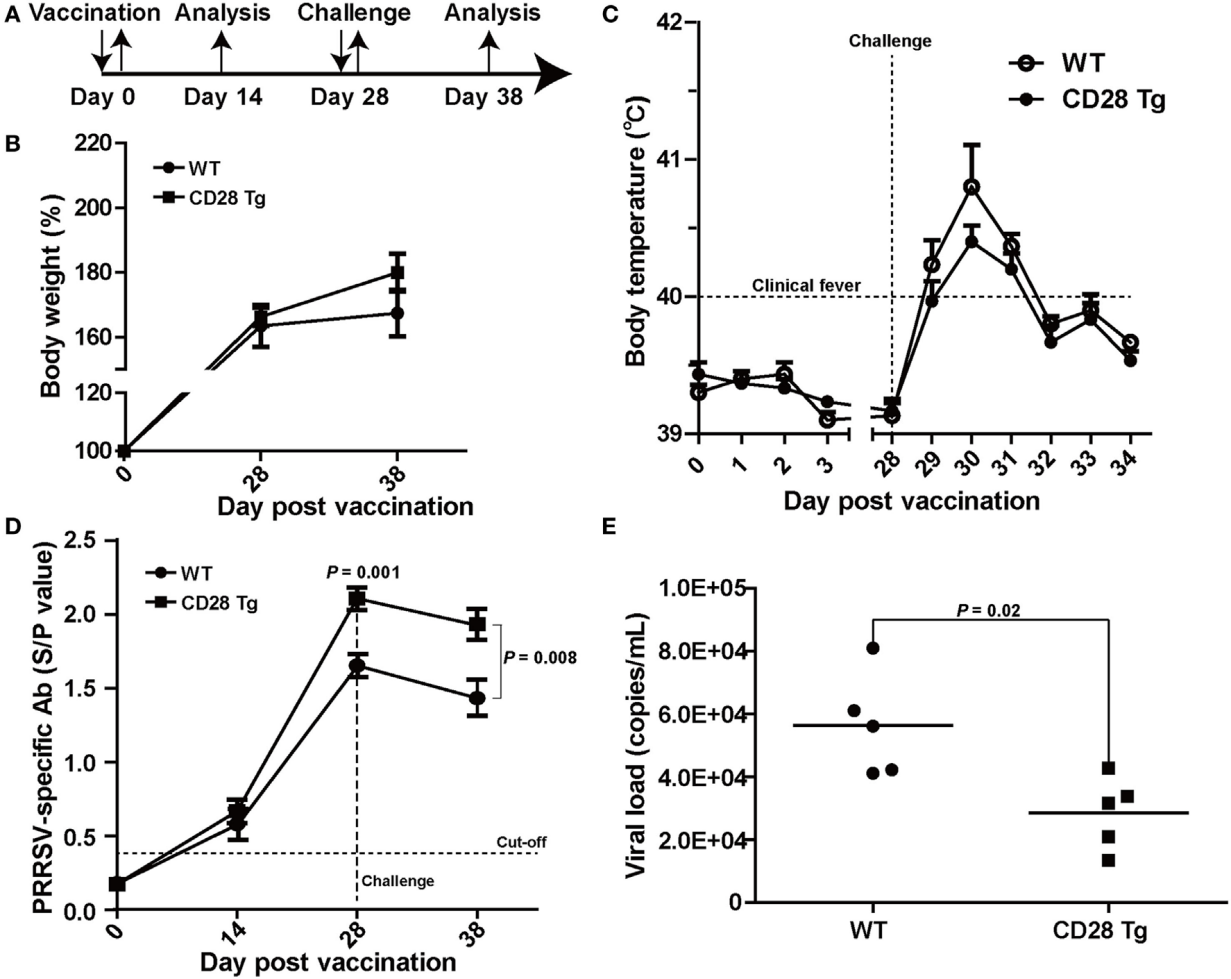
CD28 Tg pigs show improved specific antibody response, reducing body weight loss, clinical fever and viral load during porcine reproductive and respiratory syndrome virus (PRRSV) infection. **(A)** Time line of PRRSV vaccination and challenge experiments. Five male transgenic and five male wild-type pigs 4 weeks old were used in the experiments. **(B)** Body weight changes in WT and Tg pigs over the course of the experiment. **(C)** The rectal temperature of all piglets was monitored daily postvaccination and challenge. **(D)** PRRSV-specific antibody is expressed as S/P ratios measured by enzyme-linked immunosorbent assay (ELISA). S/P ratios < 0.4 were considered seronegative. **(E)** The viral loads in serum samples of WT and Tg pigs were measured by quantitative real-time PCR (WT, wild-type pigs; CD28 Tg, CD28 transgenic pigs). *P* < 0.05 was considered statistically significant.

To test whether CD28 overexpression could protect the piglets from PRRSV infection, piglets in both WT and Tg groups were vaccinated IM with a modified live PRRSV vaccine (JXA1-R, China Animal Husbandry Industry Co., Ltd., Beijing, China) and challenged with a 50-fold dose of the same strain at day 28 (Figure 3A). First, we observed that CD28 overexpression enhanced host global responses to PRRSV vaccination. All piglets in both groups exhibited a similar increase in body weight after PRRSV vaccination (Figure 3B). Moreover, a specific antibody response to PRRSV vaccine was detected in the serum until 14 days after inoculation. Compared to pigs in the WT group, Tg pigs showed a significant increase in PRRSV-specific antibody in serum at day 28 postvaccination (*P* = 0.001, Figure 3D), indicating that CD28 overexpression amplified the vaccine efficacy for eliciting a robust viral-specific antibody response.

We assessed whether the boosted immune response through CD28 signaling could protect the vaccinated pigs from challenge with a 50-fold dose of PRRSV vaccine strain. After challenge, all piglets showed a poor growth performance along with clinical fever (>40°C). It was noted that WT pigs had more growth retardation and higher fever than pigs in the Tg group (Figures 3B,C). The clinical fever gradually subsided in the following 4 days in both groups. Strikingly, a significant amount of antibody was still present in both Tg and WT pigs 10 days after challenge (*P* = 0.008, Figure 3D). Moreover, virus levels in blood of Tg pigs were lower than those in WT at day 10 postchallenge (Figure 3E). Overall, CD28 overexpression improved PRRSV-specific antibody response and reduced body weight loss, clinical fever and viral load during PRRSV challenge.

### CD28 Tg Pigs Showed Increased Frequencies of CD4^+^ T Cells after Vaccination and of CD8^+^ T Cells after Challenge

**Figure 4:**
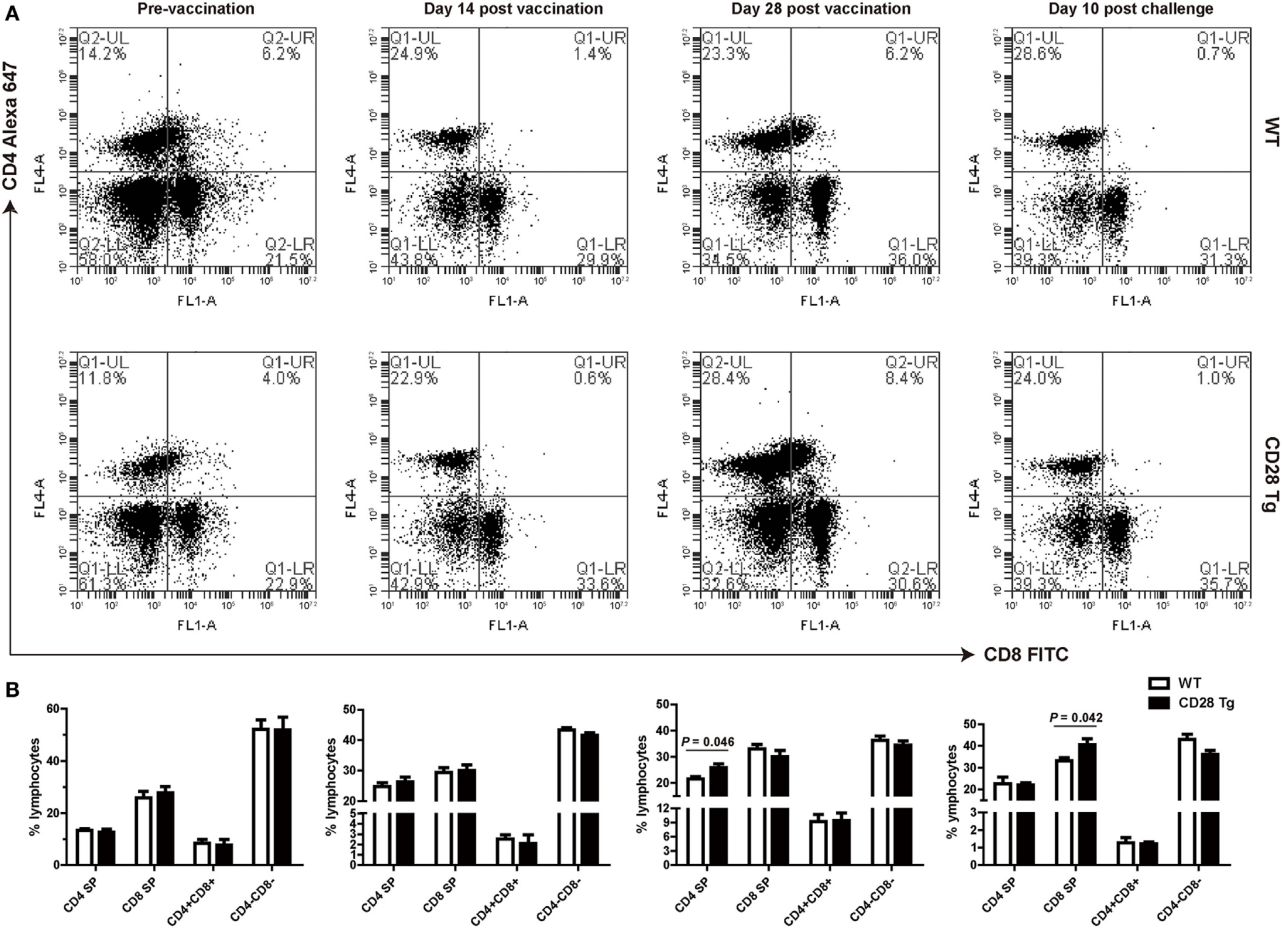
Peripheral blood lymphocyte populations of WT and CD28 Tg pigs. **(A)** Flow cytometry analysis of CD4^+^ and CD8^+^ fractions of lymphocytes in peripheral blood (WT, wild-type pigs; CD28 Tg, CD28 transgenic pigs). **(B)** Summary of CD4^+^ and CD8^+^ lymphocyte population in flow cytometry analysis. *P* < 0.05 was considered statistically significant.

We compared frequencies of CD4^+^ single-positive (SP), CD8^+^ SP, and CD4^+^CD8^+^ double-positive (DP) T cells in peripheral blood of both Tg and WT pigs at day 0 (prevaccination), days 14 and 28 postvaccination, and day 10 postchallenge by flow cytometry (Figures 4A,B). It is notable that there was no significant difference neither in CD4 nor in CD8 lymphocytes between WT and Tg groups until day 28 postvaccination (Figures 4A,B). A remarkably increased population of CD4^+^ SP cells was present in Tg pigs, responding to PRRSV vaccination at day 28 (*P* = 0.046, mean 28% of gated lymphocytes). CD8^+^ cell populations were not affected on either group during PRRSV vaccination (Figures 4A,B). By contrast, after challenge, Tg pigs had a significantly higher proportion of CD8^+^ SP cells than WT pigs (*P* = 0.042, Figures 4A,B). This means that the CD4^+^ T cell population was boosted in Tg pigs resulting from the PRRSV vaccination, and that the CD8^+^ T cell response was dominant during challenge. The population of CD4^+^CD8^+^ DP T cells did not change in response to vaccination, but decreased after challenge.

### Virus Neutralization Antibody Response to Homologous PRRSV Strains Is Strong in CD28 Tg Pigs

**Figure 5:**
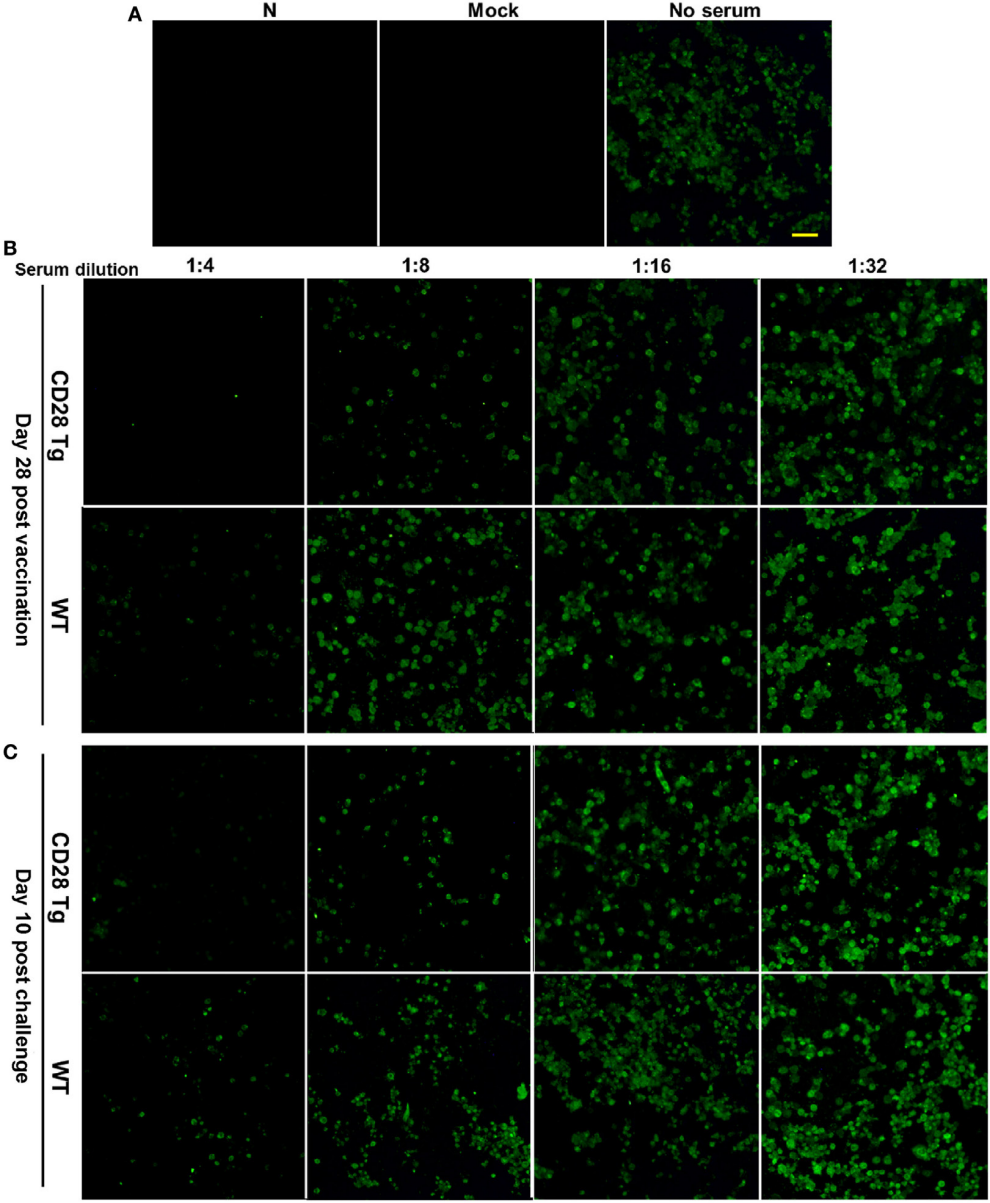
Representative pictures showing virus-neutralizing antibody response in the plasma samples of porcine reproductive and respiratory syndrome virus (PRRSV)-infected pigs. **(A)** 100 TCID_50_ of virus was used in the virus neutralization (VN) assay, which showed appreciable quantity of infected cells by immunofluorescence assay (IFA) at 24 h postinfection (N, PAMs used as negative control; Mock, mock infected; No serum, JXA-1 used as virus control. Scale bar = 50 µm.). **(B)** The VN antibody titers in the plasma samples collected at day 28 postvaccination were up to 8 in Tg pigs, whereas those in WT were 4. **(C)** VN antibody titers in plasma samples collected at day 10 postchallenge were tested again, and similar results were shown here. WT, wild-type pigs; CD28 Tg, CD28 transgenic pigs.

Virus neutralization antibody titers against PRRSV in infected serum samples were quantified by IFA. For this assay, 100 TCID_50_ of PRRSV in each well were used showing a substantial amount of infected cells 24 h postinfection (Figure 5A). Compared to WT pigs, Tg pigs exhibited a stronger anamnestic neutralizing antibody response to JXA-1 with a titer of 8 (Figures 5B,C).

### Interferon-γ Secretion by CD8^+^ T Cells Was Augmented in CD28 Tg Pigs

**Figure 6:**
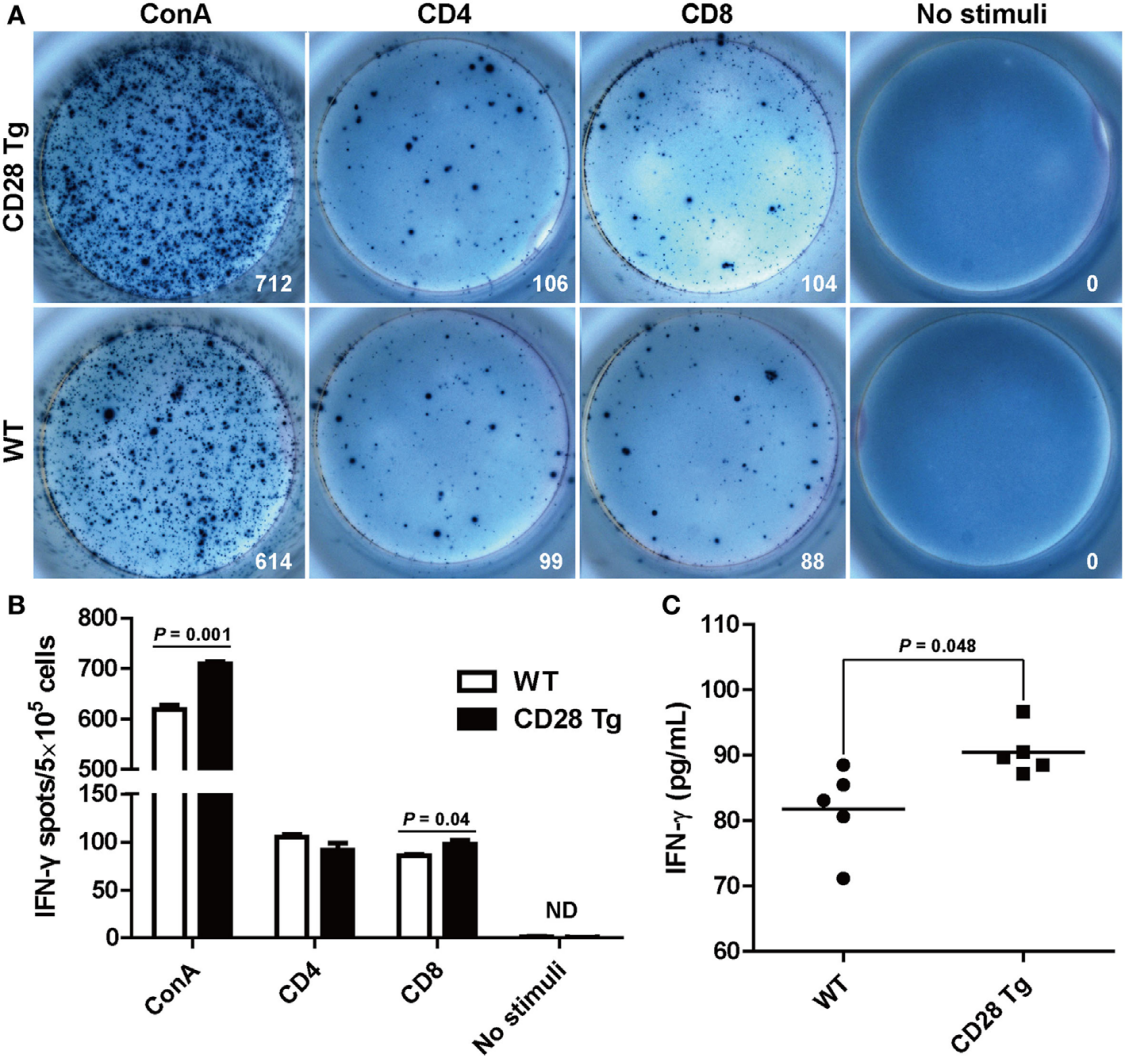
Interferon-γ (IFN-γ) secretion by peripheral blood lymphocytes in the ELISPOT assay and enzyme-linked immunosorbent assay (ELISA). **(A)** Peripheral blood mononuclear cells (PBMCs, 5 × 10^5^ cells/well) from transgenic and wild-type pigs were stimulated with CD4^+^ or CD8^+^ epitopes of porcine reproductive and respiratory syndrome virus (PRRSV) for 24 h with concanavalin A (ConA) or bovine serum albumin-coated beads, acting as positive and negative control, respectively. Wells containing PBMC alone served as controls. Samples were tested in triplicates. Images from the plate scan generated by the ImmunoSpot analyzing system are shown. Average numbers of spots per well are indicated. **(B)** Summary of the spots per well in IFN-γ ELISPOT assay. **(C)** Results shown are the concentrations of IFN-γ in serum samples of wild-type (WT) and Tg pigs (ND, not detected). *P* < 0.05 was considered statistically significant.

After observing the increase in the CD8^+^ T cell population responding to PRRSV challenge, we measured the frequency of PRRSV-specific IFN-γ secreting cells in the PBLs, which has been used as indicator of cell-mediated immunity (31). PBLs collected from Tg and WT pigs at day 38 were restimulated *in vitro* with purified ConA, CD4^+^ T cell immunodominant epitope and CD8^+^ T cell immunodominant epitope. IFN-γ response to CD8 epitope in Tg pigs was significantly higher (*P* = 0.04) than in WT pigs: between 91 and 106 spots/5 × 10^5^ cells in Tg pigs vs. 84–91 spots/5 × 10^5^ cells found in WT pigs (Figures 6A,B). There was a similar IFN-γ response to the stimulation with CD4 epitope in both groups (Figures 6A,B). The secretion of IFN-γ was also confirmed in sera by ELISA. We also found that Tg pigs had a significantly higher concentration of IFN-γ in serum samples than WT pigs (*P* = 0.048, Figure 6C). This means that PRRSV-specific CD8^+^ T cell-mediated IFN-γ secretion during PRRSV challenge was significantly enhanced in Tg pigs.

## Discussion

In this study, we generated Tg pigs expressing an extra copy of CD28 and evaluated the effect of the enhanced costimulatory signaling on T cell responses following PRRSV vaccination and challenge. We found that CD28 overexpression elicited an efficient immune response to PRRSV vaccination that can reduce the viral load in blood during challenge by improving the neutralizing antibody response and antiviral cytokine IFN-γ secretion. The role of CD28 on the enhancement of T cell-mediated immune responses suggests its potential for preventing infectious diseases.

Previous studies have raised safety concerns regarding genetic modification (36–38). Therefore, it is important to examine any potential risks derived from transgenesis. In this study, at least five founder lines of the CD28 Tg pigs were generated. The extra copy of the porcine CD28 gene was specifically integrated into the *Rosa26* locus with CRISPR/Cas9-mediated homologous recombination. In addition, the pigs had normal weights at birth and developed well under normal husbandry conditions, without any abnormalities.

Porcine reproductive and respiratory syndrome virus is still a global threat and will continue to pose challenges to pig farming because of its easy transmission, antigenic shift and drift and the limited efficacy of current vaccines and antiviral drugs (23, 39). Although deletion of CD163, a fusion receptor for PRRSV, has been previously described, resulting in a significant resistance to the virus infection, the pig could still be susceptible to other pathogens (22, 40). It is now clear that CD28 (a broad-spectrum, immunity-promoting molecule)-mediated costimulatory signaling has a crucial role in regulating T cell activation, subset differentiation, effector function, and survival (41). As expected, the forced overexpression of CD28 gene in genetically modified pigs did promote the PRRSV-specific neutralizing antibody production and IFN-γ secretion, as shown in this study.

Previous studies have shown that antibodies to PRRSV appear around 10 days after infection (26). We found that PRRSV-specific antibody in both Tg and WT groups was absent at day 7 (data not shown), but was detectable 14 days postvaccination. Increased neutralizing antibody levels were detected in CD28 Tg pig sera resulting from immunization with modified live strains of PRRSV (JXA1-R). Antibody neutralization was still evident in Tg pigs 10 days after challenge. These data indicate that CD28-mediated costimulation is necessary for eliciting a humoral immune response.

Cellular immune response, which is essential for the elimination of intracellular pathogens such as PRRSV, could protect swine from becoming infected and shedding viruses (42). Previous studies have shown that CD8^+^ T cell response is the dominant immune response to PRRSV challenge (26, 33, 43, 44). We found a remarkable increase in CD8^+^ T cell population in Tg pigs compared to WT pigs after challenge. We also found high levels of IFN-γ-secreting CD8^+^ T cells as well as high IFN-γ concentration in sera of Tg pigs. This means that CD28 overexpression considerably enhances CD8^+^ T cell-mediated cellular immune response contributing to the clearance of PRRSV in blood after challenge.

An excess of CD8 to CD4 T cells in blood is frequently observed in swine, in contrast to less CD8^+^ T cell population in human and mice (43, 45, 46). Notably, PRRSV does not alter the large majority of T cell subpopulations, including CD4^+^ SP, CD8^+^ SP, CD4^+^CD8^+^ DP, and CD4^−^CD8^−^ DN T cells (43, 47, 48). However, CD28 transgene showed a preponderance of CD4^+^ T cells in response to PRRSV vaccination, resulting in a strong VN antibody response. Moreover, PRRSV-specific IFN-γ-producing CD8^+^ T cells, another potential indicator of protective cellular immunity against PRRSV, also increased in CD28 Tg pigs. These increases indicate that CD28 plays a critical role in the induction of host humoral and cellular immune responses.

In conclusion, for the first time, we report the generation of CD28 Tg pigs, in which forced overexpression of porcine-derived CD28 gene induced protective immune responses against PRRSV infection. The role of CD28 costimulation in immune responses of transgenic pigs to a broad spectrum of pathogens such as bacteria and protists will also be examined in our future studies. Our findings reveal a successful translation of confirmed immunological findings into farm animal breeding: The broad-spectrum, immunity-promoting molecule CD28 combined with the technical use of transgene, produce a farm animal with improved immune responses to vaccination and pathogen infection.

## Ethics Statement

All animal experiments were performed in strict accordance with the Guide for the Care and Use of Laboratory Animals of the Ministry of Science and Technology of China and were approved by the Institutional Animal Care and Use Committee of China Agricultural University (certificate of the Beijing Laboratory Animal employee, ID: 18086). All efforts were made to minimize animal suffering.

## Author Contributions

The study was conceived and experiments designed by XS, GH, and XL. The experiments were performed by GH, LD, and XT. Data were analyzed and interpreted by XS, XL, GH, and LD. XS, QL, XH, WF, and LZ provided reagents, materials, or analysis tools. GH drafted the article and all authors revised the manuscript.

## Conflict of Interest Statement

The authors declare that the research was conducted in the absence of any commercial or financial relationships that could be construed as a potential conflict of interest.

## References

[1] Soares MP Teixeira L Moita LF. Disease tolerance and immunity in host protection against infection. Nat Rev Immunol (2017) 17(2):83–96. 10.1038/nri.2016.136 28044057

[2] Buchholz VR Schumacher TNM Busch DH. T cell fate at the single-cell level. Annu Rev Immunol (2016) 34(1):65–92. 10.1146/annurev-immunol-032414-112014 26666651

[3] Rosendahl Huber S van Beek J de Jonge JR Luytjes W van Baarle D. T cell responses to viral infections – opportunities for peptide vaccination. Front Immunol (2014) 5:171. 10.3389/fimmu.2014.00171 24795718 PMC3997009

[4] Brownlie RJ Zamoyska R. T cell receptor signalling networks: branched, diversified and bounded. Nat Rev Immunol (2013) 13(4):257–69. 10.1038/nri3403 23524462

[5] Chakraborty K Weiss A. Insights into the initiation of TCR signaling. Nat Immunol (2014) 15(9):798–807. 10.1038/ni.2940 25137454 PMC5226627

[6] Chen L Flies DB. Molecular mechanisms of T cell co-stimulation and co-inhibition. Nat Rev Immunol (2013) 13(4):227–42. 10.1038/nri3405 23470321 PMC3786574

[7] Zhu Y Yao S Chen L. Cell surface signaling molecules in the control of immune responses: a tide model. Immunity (2011) 34(4):466–78. 10.1016/j.immuni.2011.04.008 21511182 PMC3176719

[8] Bishop GA B cell-T cell interaction: antigen bridge to antigen presentation. Nat Rev Immunol (2016) 16(8):467–467. 10.1038/nri.2016.82 27396445

[9] Tan PH Yates J Xue S Jordan WJ Dong R Ritter MA Prevention of co-stimulation molecule expression using intracellular CTLA4: a novel strategy for induction of T cell anergy. Xenotransplantation (2003) 10(5):531–531.

[10] Tarte K Klein B. Dendritic cell-based vaccine: a promising approach for cancer immunotherapy. Leukemia (1999) 13(5):653–63. 10.1038/sj.leu.2401394 10374867

[11] Acuto O Michel F CD28-mediated co-stimulation: a quantitative support for TCR signalling. Nat Rev Immunol (2003) 3(12):939–51. 10.1038/nri1248 14647476

[12] Alegre M Thompson CB Frauwirth KA. T-cell regulation by CD28 and CTLA-4. Nat Rev Immunol (2001) 1(3):220–8. 10.1038/35105024 11905831

[13] Sharpe H Freeman GJ The B7–CD28 superfamily. Nat Rev Immunol (2002) 2(2):116–26. 10.1038/nri727 11910893

[14] Abe R Vandenberghe P Craighead N Smoot DS Lee KP June CH Distinct signal-transduction in mouse CD4(+) and CD8(+) splenic T-cells after CD28 receptor ligation. J Immunol (1995) 154(3):985–97. 7822814

[15] Salomon B Lenschow DJ Rhee L Ashourian N Singh B Sharpe A B7/CD28 costimulation is essential for the homeostasis of the CD4(+)CD25(+) immunoregulatory T cells that control autoimmune diabetes. Immunity (2000) 12(4):431–40. 10.1016/S1074-7613(00)80195-8 10795741

[16] Hamann D Baars P Rep M Hooibrink B KerkhofGarde SR Klein MR Phenotypic and functional separation of memory and effector human CD8(+) T cells. J Exp Med (1997) 186(9):1407–18. 10.1084/jem.186.9.1407 9348298 PMC2199103

[17] Boomer JS Green JM. An enigmatic tail of CD28 signaling. Cold Spring Harb Perspect Biol (2010) 2:a0024368. 10.1101/cshperspect.a002436 20534709 PMC2908766

[18] Janardhan SV Praveen K Marks R Gajewski TF Evidence implicating the ras pathway in multiple CD28 costimulatory functions in CD4(+) T cells. PLoS One (2011) 6:e249319 10.1371/journal.pone.0024931 PMC3176298 21949793

[19] Linterman MA Denton AE Divekar DP Zvetkova I Kane L Ferreira C CD28 expression is required after T cell priming for helper T cell responses and protective immunity to infection. Elife (2014) 3:e03180. 10.7554/eLife.03180 25347065 PMC4241536

[20] Frohlich M Gogishvili T Langenhorst D Luhder F Hunig T. Interrupting CD28 costimulation before antigen rechallenge affects CD8(+) T-cell expansion and effector functions during secondary response in mice. Eur J Immunol (2016) 46(7):1644–55. 10.1002/eji.201546232 27122236

[21] Li Y Zhou L Zhang J Ge X Zhou R Nsp9 and Nsp10 contribute to the fatal virulence of highly pathogenic porcine reproductive and respiratory syndrome virus emerging in China. PLoS Pathog (2014) 10:e1004216 10.1371/journal.ppat.1004344 24992286 PMC4081738

[22] Burkard C Lillico SG Reid E Jackson B Mileham AJ Ait-Ali T Precision engineering for PRRSV resistance in pigs: macrophages from genome edited pigs lacking CD163 SRCR5 domain are fully resistant to both PRRSV genotypes while maintaining biological function. PLoS Pathog (2017) 13(2):e1006206. 10.1371/journal.ppat.1006206 28231264 PMC5322883

[23] Renukaradhya GJ Meng X Calvert JG Roof M Lager KM Live porcine reproductive and respiratory syndrome virus vaccines: current status and future direction. Vaccine (2015) 33(33):4069–80. 10.1016/j.vaccine.2015.06.092 26148878

[24] Huang C Zhang Q Feng W. Regulation and evasion of antiviral immune responses by porcine reproductive and respiratory syndrome virus. Virus Res (2015) 202:101–11. 10.1016/j.virusres.2014.12.014 25529442 PMC7132515

[25] Yoo D Song C Sun Y Du Y Kim O Liu H. Modulation of host cell responses and evasion strategies for porcine reproductive and respiratory syndrome virus. Virus Res (2010) 154(1–2):48–60. 10.1016/j.virusres.2010.07.019 20655963 PMC7114477

[26] Meier WA Galeota J Osorio FA Husmann RJ Schnitzlein WM Zuckermann FA Gradual development of the interferon-γ response of swine to porcine reproductive and respiratory syndrome virus infection or vaccination. Virology (2003) 309(1):18–31. 10.1016/S0042-6822(03)00009-6 12726723

[27] Chung YG Eum JH Lee JE Shim SH Sepilian V Hong SW Human somatic cell nuclear transfer using adult cells. Cell Stem Cell (2014) 14(6):777–80. 10.1016/j.stem.2014.03.015 24746675

[28] Kim HS Kwang J Yoon IJ Joo HS Frey ML Enhanced replication of porcine reproductive and respiratory syndrome (PRRS) virus in a homogeneous subpopulation of MA-104 cell line. Arch Virol (1993) 133:477–83. 10.1007/BF01313785 8257302

[29] Kimura M Stone RC Hunt SC Skurnick J Lu X Cao X Measurement of telomere length by the Southern blot analysis of terminal restriction fragment lengths. Nat Protoc (2010) 5(9):1596–607. 10.1038/nprot.2010.124 21085125

[30] Meloche S Pouyssegur J. The ERK1/2 mitogen-activated protein kinase pathway as a master regulator of the G1- to S-phase transition. Oncogene (2007) 26(22):3227–39. 10.1038/sj.onc.1210414 17496918

[31] Skern R Frost P Nilsen F. Relative transcript quantification by quantitative PCR: roughly right or precisely wrong? BMC Mol Biol (2005) 6:10. 10.1186/1471-2199-6-10 15854230 PMC1090581

[32] Joshi MU Pittman HK Haisch CE Verbanac KM. Real-time PCR to determine transgene copy number and to quantitate the biolocalization of adoptively transferred cells from EGFP-transgenic mice. Biotechniques (2008) 45(3):247. 10.2144/000112913 18778249

[33] Zuckermann FA Garcia EA Luque ID Christopher-Hennings J Doster A Brito M Assessment of the efficacy of commercial porcine reproductive and respiratory syndrome virus (PRRSV) vaccines based on measurement of serologic response, frequency of gamma-IFN-producing cells and virological parameters of protection upon challenge. Vet Microbiol (2007) 123(1–3):69–85. 10.1016/j.vetmic.2007.02.009 17376612

[34] Ostrowski M Galeota JA Jar AM Platt KB Osorio FA Lopez OJ Identification of neutralizing and nonneutralizing epitopes in the porcine reproductive and respiratory syndrome virus GP5 ectodomain (vol 76, pg 4241, 2002). J Virol (2002) 76(13):6863–6863. 10.1128/JVI.76.13.6863.2002 PMC155073 11932389

[35] Mokhtar H Pedrera M Frossard J Biffar L Hammer SE Kvisgaard LK The non-structural protein 5 and matrix protein are antigenic targets of T cell immunity to genotype 1 porcine reproductive and respiratory syndrome viruses. Front Immunol (2016) 7:40. 10.3389/fimmu.2016.00040 26909080 PMC4755262

[36] Boudreau RL Martins I Davidson BL. Artificial microRNAs as siRNA shuttles: improved safety as compared to shRNAs *in vitro* and *in vivo*. Mol Ther (2009) 17(1):169–75. 10.1038/mt.2008.231 19002161 PMC2834985

[37] Cao W Hunter R Strnatka D McQueen CA Erickson RP. DNA constructs designed to produce short hairpin, interfering RNAs in transgenic mice sometimes show early lethality and an interferon response. J Appl Genet (2005) 46(2):217–25. 15876690

[38] Storm TA Pandey K Jopling CL Davis CR Marion P Salazar F Fatality in mice due to oversaturation of cellular microRNA/short hairpin RNA pathways. Nature (2006) 441(7092):537–41. 10.1038/nature04791 16724069

[39] Murtaugh MP Genzow M. Immunological solutions for treatment and prevention of porcine reproductive and respiratory syndrome (PRRS). Vaccine (2011) 29(46):8192–204. 10.1016/j.vaccine.2011.09.013 21925560

[40] Whitworth KM Rowland RRR Ewen CL Trible BR Kerrigan MA Cino-Ozuna AG Gene-edited pigs are protected from porcine reproductive and respiratory syndrome virus. Nat Biotechnol (2016) 34(1):20–2. 10.1038/nbt.3434 26641533

[41] Fraser JD Irving BA Crabtree GR Weiss A. Regulation of interleukin-2 gene enhancer activity by the T cell accessory molecule CD28. Science (1991) 251(4991):313–6. 10.1126/science.1846244 1846244

[42] Galliher-Beckley A Li X Bates JT Madera R Waters A Nietfeld J Pigs immunized with Chinese highly pathogenic PRRS virus modified live vaccine are protected from challenge with North American PRRSV strain NADC-20. Vaccine (2015) 33(30):3518–25. 10.1016/j.vaccine.2015.05.058 26049004

[43] Shimizu M Yamada S Kawashima K Ohashi S Shimizu S Ogawa T. Changes of lymphocyte subpopulations in pigs infected with porcine reproductive and respiratory syndrome (PRRS) virus. Vet Immunol Immunopathol (1996) 50(1–2):19–27. 10.1016/0165-2427(95)05494-4 9157683

[44] Costers S Lefebvre DJ Goddeeris B Delputte PL Nauwynck HJ Functional impairment of PRRSV-specific peripheral CD3(+)CD8(high) cells. Vet Res (2009) 40:465 10.1051/vetres/2009029 PMC2701180 19445889

[45] Zuckermann FA Gaskins HR. Distribution of porcine CD4/CD8 double-positive T lymphocytes in mucosa-associated lymphoid tissues. Immunology (1996) 87(3):493–9. 10.1046/j.1365-2567.1996.494570.x 8778039 PMC1384122

[46] Zuckermann FA Husmann RJ. Functional and phenotypic analysis of porcine peripheral blood CD4/CD8 double-positive T cells. Immunology (1996) 87(3):500–12. 8778040 PMC1384123

[47] Loving CL Osorio FA Murtaugh MP Zuckermann FA. Innate and adaptive immunity against porcine reproductive and respiratory syndrome virus. Vet Immunol Immunopathol (2015) 167(1–2):1–14. 10.1016/j.vetimm.2015.07.003 26209116 PMC7112826

[48] Lohse L Nielsen J Eriksen L Temporary CD8+ T-cell depletion in pigs does not exacerbate infection with porcine reproductive and respiratory syndrome virus (PRRSV). Viral Immunol (2004) 17:594–603. 10.1089/vim.2004.17.594 15671757

